# Liposomal clodronate selectively eliminates microglia from primary astrocyte cultures

**DOI:** 10.1186/1742-2094-9-116

**Published:** 2012-05-31

**Authors:** Hiromi Kumamaru, Hirokazu Saiwai, Kazu Kobayakawa, Kensuke Kubota, Nico van Rooijen, Kazuhide Inoue, Yukihide Iwamoto, Seiji Okada

**Affiliations:** 1 Department of Orthopedic Surgery, Kyushu University, 3-1-1 Maidashi, Higashi-ku, Fukuoka 812-8582, Japan; 2 Department of Advanced Medical Initiatives, Graduate School of Medical Sciences, Kyushu University, 3-1-1 Maidashi, Higashi-ku, Fukuoka 812-8582, Japan; 3 Department of Molecular Cell Biology, Vrije University Medical Center, Amsterdam, 1081 HV, The Netherlands; 4 Department of Molecular and System Pharmacology, Graduate School of Pharmaceutical Sciences, Kyushu University, 3-1-1 Maidashi, Higashi-ku, Fukuoka 812-8582, Japan

**Keywords:** Astrocytes, Liposomal clodronate, Microglia, Inflammation

## Abstract

**Background:**

There is increasing interest in astrocyte biology because astrocytes have been demonstrated to play prominent roles in physiological and pathological conditions of the central nervous system, including neuroinflammation. To understand astrocyte biology, primary astrocyte cultures are most commonly used because of the direct accessibility of astrocytes in this system. However, this advantage can be hindered by microglial contamination. Although several authors have warned regarding microglial contamination in this system, complete microglial elimination has never been achieved.

**Methods:**

The number and proliferative potential of contaminating microglia in primary astrocyte cultures were quantitatively assessed by immunocytologic and flow cytometric analyses. To examine the utility of clodronate for microglial elimination, primary astrocyte cultures or MG-5 cells were exposed to liposomal or free clodronate, and then immunocytologic, flow cytometric, and gene expression analyses were performed. The gene expression profiles of microglia-eliminated and microglia-contaminated cultures were compared after interleukin-6 (IL-6) stimulation.

**Results:**

The percentage of contaminating microglia exceeded 15% and continued to increase because of their high proliferative activity in conventional primary astrocyte cultures. These contaminating microglia were selectively eliminated low concentration of liposomal clodronate. Although primary microglia and MG-5 cells were killed by both liposomal and free clodronate, free clodronate significantly affected the viability of astrocytes. In contrast, liposomal clodronate selectively eliminated microglia without affecting the viability, proliferation or activation of astrocytes. The efficacy of liposomal clodronate was much higher than that of previously reported methods used for decreasing microglial contamination. Furthermore, we observed rapid tumor necrosis factor-α and IL-1b gene induction in conventional primary astrocyte cultures after IL-6 stimulation, which was due to the activation of the Janus kinase/signal transducer and activator of the transcription pathway in contaminating microglia.

**Conclusions:**

Because contaminating microglia could result in erroneous data regarding the pro-inflammatory properties of astrocytes, astrocyte biology should be studied in the absence of microglial contamination. Our simple method will be widely applicable to experimental studies of astrocyte biology and provide clues for understanding the role of astrocytes in neural development, function and disease.

## Background

Astrocytes comprise the majority of cells in the central nervous system (CNS). Although astrocytes were originally defined as gap fillers for neuronal networks, they have been found to play prominent roles in normal CNS functions, such as energy metabolism, neurotransmission, maintenance of blood–brain barrier, extracellular ion homeostasis and cerebrovascular regulation [1,2]. In pathological conditions, they also participate in neuroinflammation and tissue repair [2-5]. Much of this knowledge was obtained through experimental studies with rodent primary astrocyte cultures. Because primary astrocyte cultures are convenient and easy to establish, they are most commonly used for assessing astrocyte biology [6]. In brief, these cultures are prepared by dissociation of cells of the dissected brain or spinal cord and plating of the dissociated cells on dishes. With regard to the detailed protocol for this method, several minor modifications have been incorporated since the original method was described [7-10]. However, these modifications result in cultures in which astrocytes are the predominant cells, but never comprise 100% of the cells in these preparations (approximately 90% to 95%) [6].

Microglia, the resident tissue macrophages of CNS, are frequent contaminants of primary astrocyte cultures [6]. Microglial contamination has been often underestimated because relatively small numbers of microglia were observed in these cultures [6]. However, recent studies suggested that the presence of microglia in primary astrocyte cultures largely affects astrocyte responses *in vitro*[11-13]. This will be especially problematic when sensitive biochemical methods are employed, such as high-through-put next-generation sequencing technology [14]. In the CNS, microglia provides first line of host defense despite that they comprise a relatively small proportion of CNS cells. Microglia respond rapidly to pathological alterations [15,16] and regulate responses of other cells, including astrocytes [17-19]. Similarly, astrocytes mediate responses, proliferation and activation of microglia by releasing various cytokines or ATP [15,20]. Because these complex cell–cell interactions confound the understanding of astrocyte function [6], astrocytes need to be investigated in microglia-free conditions to accurately assess their biology.

Clodronate belongs to a family of bisphosphonates and is widely used to treat osteolytic diseases and osteoporosis because of its inhibitory effect on osteoclasts [21]. This drug is known to induce apoptosis in macrophages or macrophage-like cells [22] and is used to eliminate these cells *in vivo*[23]. Because freely dissolved clodronate (free clodronate) is a highly hydrophilic molecule that hardly crosses cellular phospholipid membranes [21], liposome-mediated intracellular delivery of clodronate has been developed for macrophage elimination [24]. Liposomes are artificially prepared lipid vesicles consisting of concentric phospholipid bilayers entrapping aqueous compartments that are used to encapsulate hydrophilic molecules to dissolve them in aqueous solutions [25]. Liposome-encapsulated clodronate (liposomal clodronate) is specifically phagocytized by macrophages. After phagocytosis, lysosomal action disrupts the fatty bilayers of the liposome, and free clodronate is then released into the cell, causing irreversible functional damage and apoptosis [26]. Selective elimination of macrophages by liposomal clodronate was reported *in vitro*[22,27].

Since recent study demonstrated microglial death by liposomal clodronate in the brain [28], we hypothesized that liposomal clodronate can eliminate microglia in primary astrocyte cultures. In this study, we demonstrated selective microglial elimination from primary astrocyte cultures using liposomal clodronate without affecting the viability, proliferation or activation of astrocytes. Furthermore, by comparing the gene expression profiles of microglia-eliminated and microglia-contaminated primary astrocyte cultures after interleukin-6 (IL-6) stimulation, we found that contaminating microglia induced deceptive up-regulation of tumor necrosis factor alpha (TNF-α) and interleukin (IL)-1β genes in conventional primary astrocyte cultures through the activation of the Janus kinase/signal transducer and activator of the transcription (JAK/STAT) pathway, thus highlighting the importance of microglial elimination from primary astrocyte cultures. Using microglia-eliminated cultures, functional aspects of astrocytes can be accurately studied without complex interactions in response to specific molecules. Our simple and useful method will be widely applicable to experimental studies of astrocyte biology.

## Material and methods

### Preparation of primary astrocyte cultures

Conventional primary astrocyte cultures were prepared from C57BL/6 mice as described previously [6,29,30]. In brief, after removal of the meninges, postnatal day 3 (P3) mouse brain tissues were minced and incubated in a rocking water bath at 37 °C for 30 minutes in Dulbecco’s modified Eagle’s medium (DMEM; Invitrogen, Carlsbad, CA, USA) in the presence of 300 g/mL DNase I (Sigma-Aldrich, St. Louis, MO, USA) and 0.25% trypsin (Sigma-Aldrich). Enzyme-digested dissociated cells were triturated with 0.25% fetal bovine serum (FBS), washed and centrifuged at 300 × *g* for five minutes. The pellet was resuspended in DMEM, passed through a 30-μm nylon mesh, washed, and centrifuged at 300 × *g* for five minutes. Following dilution with astrocyte-specific medium (DMEM containing 10% FBS, 0.2 mM l-glutamine, and 1% penicillin–streptomycin), the cells were plated on poly-l-lysine–coated culture dishes at the density of 1.0 × 10^5^ cells/cm^2^ and allowed to adhere for one day in a humidified CO_2_ incubator at 37 °C. Next, non-adherent cells were removed, and fresh astrocyte-specific medium was added. Adherent cells were maintained in astrocyte-specific medium for seven days with a medium change every two to three days [6]. For passage, monolayers were rinsed with phosphate-buffered saline (PBS) and then dislodged by trypsinization (0.25% trypsin and 0.02% ethylenediaminetetraacetic acid) for three minutes at 37 °C and plated on poly-l-lysine-coated dishes at the density of 5.0 × 10^4^ cells/cm^2^. Passaged astrocyte cultures between three and five weeks *in vitro* were used throughout, unless otherwise specified. All experimental manipulations were approved by the Ethics Committee on Animal Experiment in the Faculty of Medicine, Kyushu University, and conducted under the control of the Guidelines for Animal Experimentation.

### Conventional shake-off method

Primary astrocyte cultures were thoroughly agitated in an orbital incubator shaker at 350 rpm and 37 °C for 12 h on Day 7 after their establishment. Immediately after agitation, all cells suspended in the culture medium were discarded, and attached cells were sub-cultured in astrocyte-specific medium [6].

### Preparation of liposomal clodronate

Liposomal clodronate was prepared as previously described [25,31]. In brief, 4.30 mL phosphatidylcholine solution was added to 4.00 mL cholesterol solution in a 0.5 liter round bottom flask. The ethanol was removed by low vacuum (58 mbar) rotary (150 rpm) evaporation at 40 °C. The condensed ethanol was removed by aerating the flask three times. The phospholipid film was dispersed in 20 mL clodronate solution (for liposomal clodronate) or 20 ml PBS (for empty liposomes) by gentle rotation at room temperature. The suspension was kept at room temperature for about two hours and then the solution was gently shaken. The suspension was put in a 50 ml plastic tube and sonicated in a water bath (55 kHz) for three minutes. The suspension was kept at room temperature for two hours. Before using the liposomal clodronate, the non-encapsulated clodronate was removed by centrifuging the liposomes at 24,000 × g and 10 °C for 60 minutes. The clodronate liposomes will form a white band at the top of the suspension, whereas the suspension itself will be nearly clear. Carefully remove the clodronate solution under the white band of liposomes with a 10 ml pipet. The liposomes was resuspended in approximately 45 ml PBS and washed four to five times. The final liposome pellet was resuspended in PBS and adjusted to a final volume of 20.0 mL. The suspension was gently shaken before dispensing to achieve a homogeneous distribution of the liposomes in suspension. Clodronate in PBS was used as the control for liposomal clodronate, and clodronate disodium salt (Calbiochem, La Jolla, CA, USA) was used as free clodronate.

### Microglial cell line culture

The MG-5 murine microglial cell line (kindly provided by Dr Kohsaka S., Department of Neurochemistry, National Institute of Neuroscience) was cultured as described previously [32,33]. Conditioned medium from the supernatant of murine astrocyte cell line A1 cells (kindly provided by Dr Kohsaka S.) cultured overnight in DMEM containing 10% FBS and penicillin-streptomycin (20 U/mL) was used as the culture medium for MG-5 cells. The BV-2 murine microglial cell line (kindly provided by Dr. Biber K., Department of Medical Physiology, University Medical Center Groningen, University of Groningen) was cultured in DMEM with 5% FBS, 2 mM l-glutamine, and 1% penicillin–streptomycin, as described previously [34,35].

### Immunocytologic analysis

Cultures were fixed for 15 minutes in cold 4% paraformaldehyde (PFA) in PBS at room temperature and post-fixed in methanol at −20 °C for 15 minutes. After being washed three times with PBS, fixed samples were permeabilized in 0.01% Triton-X in PBS for 30 minutes and blocked with 5% normal goat serum for 1 h at 4 °C. Primary antibodies in the blocking solution were applied overnight at 4 °C. Astrocytes were identified using the rabbit anti-glial fibrillary acidic protein antibody (anti-GFAP; 1:1,000; Dako Cytomation, Glostrup, Denmark, rat, mouse,), rat anti-GFAP (1:1,000; Invitrogen), or mouse anti-GFAP(1:1,000; Sigma-Aldrich). Microglia were identified using rabbit anti-ionized calcium-binding adaptor molecule 1 (Iba1; 1:200; Wako, Osaka, Japan) or rat anti-CD68 (1:200, Serotec, Oxford, UK). Rabbit anti-phosphorylated-STAT3 (Tyr705) (anti-pSTAT3; 1:200, Cell Signaling Technology, Danvers, MA, USA) were used to identify the activation of JAK/STAT pathway. After rinsing, samples were incubated with Alexa Fluor secondary antibody conjugates for mouse, rabbit, or rat IgG (1:200, Invitrogen) in blocking solution for 1 h at room temperature. Nuclear counterstaining was performed using Hoechst 33342 (Invitrogen). Proliferating cells were identified using rabbit anti-Ki67 (1:1,000, Novocastra, Newcastle, UK) or rabbit anti-phosphorylated histone H3 (Ser10) (p-His-H3; 1:1,000, Upstate/Millipore, MA, USA). For propidium iodide (PI) staining, 0.1 mg/ml PI was added to the culture medium for 2 h, and the cells were fixed in 4% PFA. Fluorescent- labeled samples were coverslipped with fluorescent mounting medium (Dako Cytomation). Images were captured using the BZ-9000 digital microscope system (Keyence, Osaka, Japan) or an epifluorescent microscope (BX51; Olympus, Tokyo, Japan) equipped with a digital camera.

### BrdU incorporation assay

The proliferation of microglia was examined by adding 50 μg⁄mL 5-bromo-20-deoxyuridine (BrdU) to the culture medium and incubating for 24 h. Following fixation, samples were pre-treated with 2 N HCL at 37 °C for five minutes. Rat anti-BrdU (1: 200; Abcam, Cambridge, UK), mouse anti-GFAP, and rabbit anti-Iba1 were used to identify proliferating astrocytes and microglia. Bound antibodies were visualized using anti-rabbit, anti-mouse, and anti-rat IgG secondary antibodies with Alexa Fluor fluorescent conjugates.

### Quantitative analysis

To quantify the number of BrdU-positive cells, anti-BrdU–immunoreactive cells were counted using the BZ-9000 digital microscope and BZII-Analyzer measurement software (Keyence, Los Angeles, CA, USA). Algorithms for counting the number of BrdU-positive cells were provided by Dynamic cell count BZ-H1C software (Keyence); these algorithms selectively count immunopositive particles that range in size from 5 to 15 μm in both dimensions (X and Y) and automatically eliminate spurious particles [19].

### Flow cytometric analysis

Flow cytometric analysis was performed as previously described [36]. In brief, confluent cultures were trypsinized and resuspended in 10 mL of fresh astrocyte-specific medium on the dish. After centrifugation at 300 × g and 4 °C for two minutes, the cell pellet was resuspended in 10 mL medium. The resulting suspension was pelleted by centrifugation at 300 × g and 4 °C for 2 minutes and then incubated on ice with fluorescent antibodies for 30 minutes. Samples were stained phycoerythrin -Cy7–conjugated CD45 (eBioscience, San Diego, CA, USA), fluorescein isothiocyanate-conjugated CD11b (eBioscience), PE-conjugated Gr-1 (eBioscience), and biotin-conjugated major histocompatibility complex class II (eBioscience). Allophycocyanin-conjugated streptavidin (eBioscience) was added to label biotin-conjugated antibodies. All samples were suspended in 500 μL fluorescence-activated cell sorting (FACS) buffer (Hanks balanced salt solution, 2.5% FBS, 0.1% NaN_3_) and analyzed at the same flow rate and duration to allow comparisons among all samples using a FACSAria II flow cytometer (BD Biosciences, San Jose, CA, USA). Data were analyzed using FACSDiva software (BD Biosciences).

### Quantitative and semi-quantitative reverse transcription-polymerase chain reaction (RT-PCR)

Total RNA was isolated from primary astrocyte cultures using the RNeasy Mini kit (Qiagen, Hilden, Germany) or from FACS-purified cells using the RNeasy Micro kit (Qiagen) following the manufacturer’s protocol. For cDNA synthesis, RT was performed using the PrimeScript 1st strand cDNA Synthesis Kit (TaKaRa, Shiga, Japan). Quantitative PCR (qPCR) was performed using primers specific for the genes of interest [see Additional file 1] and SYBR Premix Dimer Eraser (TaKaRa Bio) in 20-μL reactions. Data were normalized to glyceraldehydes-3-phosphate dehydrogenase expression (GAPDH). RT-PCR was performed on a Thermocycler (Biometra, Gottingen, Germany) and products were detected by electrophoresis and SYBR Green I (Sigma-Aldrich).

### Cell stimulation

Cells were stimulated with 50 ng/mL IL-6 (R&D Systems Inc., Minneapolis, MN, USA) and 200 ng/mL soluble IL-6 receptor (R&D Systems Inc.).

### Statistical analysis

Statistical evaluations were performed using the Mann–Whitney *U*-test. For multiple comparisons between groups, the Kruskal–Wallis H test with Bonferroni’s *post hoc* correction was used. *P* <0.05 was considered statistically significant. Data in graphs are presented as the mean ± standard error of the mean (SEM).

## Results

### Contaminating microglia increase in number over time in primary astrocyte cultures

**Figure 1:**
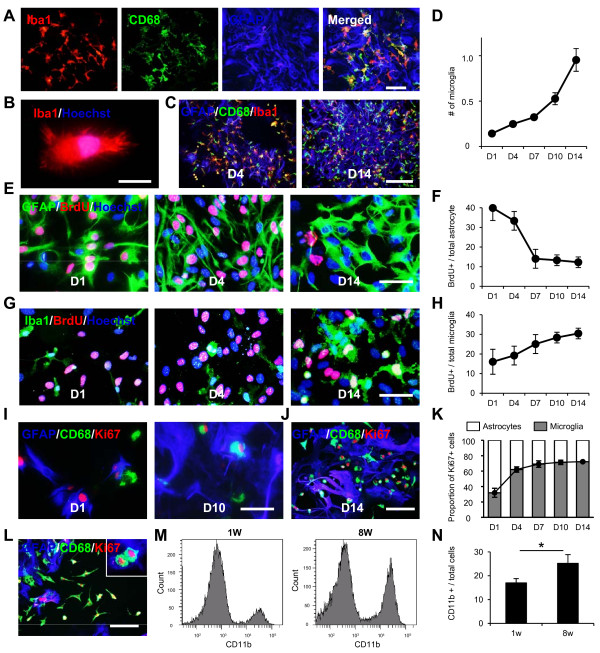
**Quantitative analysis of microglial contamination in primary astrocyte cultures.** (**A**) Triple immunostaining for primary astrocyte cultures using Iba1 (red), CD68 (green), and GFAP (blue) antibodies. Scale bar, 100 μm. (**B**) High magnification of the remaining Iba1-positive microglia after the shake-off method. Scale bar, 20 μm. (**C**) Triple immunostaining 4 and 14 days after the establishment of astrocyte primary cultures. Scale bar, 200 μm. (**D**) Quantification of the number of contaminating microglia in passaged culture. (**E**) Triple immunostaining of passaged culture using GFAP (green), BrdU (red), and Hoechst (blue) antibodies at 1, 4, and 14 days after seeding. Scale bar, 50 μm. (**F**) Changes in the number of BrdU-positive proliferating astrocytes to the total number of astrocytes over time in passaged culture. (**G**) Triple immunostaining of passaged culture using Iba1 (green) and BrdU (red) antibodies at 1, 4, and 14 days after seeding. Scale bar, 50 μm. (**H**) Changes in the number of BrdU-positive proliferating microglia to total microglia over time in passaged culture. (**I-J**) Triple immunostaining of passaged culture using GFAP (blue), CD68 (green), and Ki67 (red) antibodies at 1 and 10 days (**I**) and 14 days (**J**) after seeding. Scale bar, 50 or 200 μm. (**K**) The number of GFAP-positive astrocytes and CD11b-positive microglia in the Ki67-positive proliferating cell population. (**L**) Immunostaining of primary astrocyte cultures at 8 weeks after preparation showing Ki67-positive microglia. Scale bar, 200 μm. Inset shows high magnification image of Ki67-positive microglia. (**M**) Flow cytometric analysis at one and eight weeks after culture preparation showed increased number of CD11b-positive microglia. (**N**) Comparison of the number of the CD11b-positive population between one and eight weeks after preparation. *P <0.05, paired Student’s *t*-test. Data are presented as the mean ± SEM.

We first examined change in the number of contaminating microglia in primary astrocyte cultures over time because their proliferative activities remain elusive in this culture condition [6]. Dissociated cells from P3 mouse brains were cultured in astrocyte-specific medium at relatively low densities (5.0 × 10^4^ cells/cm^2^) [6]. Astrocytes growing in primary monolayer cultures contained a substantial number of contaminating microglia seven days after preparation, as evidenced by the high levels of Iba1 and CD68 (Figure 1A). Even after utilizing the conventional shake-off method, many microglia were observed in primary astrocyte cultures and these remaining amoeboid microglia had multiple short spinous processes (Figure 1B), suggesting strong adhesion of these cells to culture dishes. When these primary astrocyte cultures were subcultured and maintained for 14 days without passage, the number of microglia gradually increased with time, especially from 7 days after passage (Figure 1C, D), indicating the high proliferative potential of microglia in this culture. To assess their proliferation in primary astrocyte cultures, we performed the BrdU incorporation assay and Ki67 immunostaining. The BrdU incorporation assay, which labeled S-phase cells, demonstrated the presence of abundant proliferating cells in primary astrocyte cultures (Figure 1E-H). Whereas there were large number of proliferating astrocytes four days after seeding (Figure 1E), a certain number proliferating microglia were observed in primary astrocyte cultures (Figure 1G). Quantitative analysis showed that the proliferation of astrocytes was most prominent immediately after seeding and thereafter gradually decreased (Figure 1E, F). In contrast, the proliferation of microglia gradually increased over 14 days (Figure 1G, H). Quantitative analysis of cells positive for Ki67, a nuclear protein expressed in all phases of the cell cycle, confirmed the extensive proliferative potency of microglia (Figure 1I-K). Of note, the number of Ki67-positive microglia was higher than that of Ki67-positive astrocytes from seven days after seeding. We further examined the temporal changes in the number of microglia in astrocyte cultures for eight weeks with weekly passages. The proliferative potential of microglia was maintained after eight weeks of culture (Figure 1L), and the number of contaminating microglia was significantly higher at eight weeks after preparation than at one week after preparation (Figure 1M, N). These results indicate that the initially small number of contaminating microglia could dramatically increase with time in primary astrocyte cultures, thus highlighting the need for complete microglial elimination from primary astrocyte cultures.

### Liposomal and free clodronate induce apoptosis of microglia

**Figure 2:**
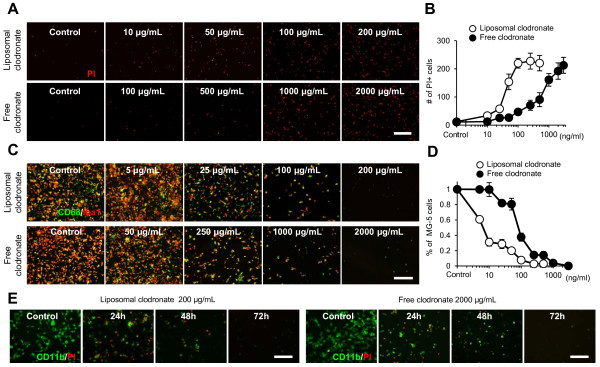
**Utility of liposomal and free clodronate for microglial elimination.** (**A**) PI staining of MG-5 cells one day after exposure to each clodronate. PI-positive cells (red) indicate dead MG-5 cells. Scale bar, 500 μm. (**B**) Effects of liposomal and free clodronate on the viability of MG-5 cells. Both forms of clodronate increased the number of dead cells in a concentration-dependent manner after 24 h of exposure. (**C**) Double immunostaining of MG-5 cells three days after exposure to each clodronate using CD68 (green) and Iba1 (red) antibodies. Scale bar, 200 μm. (**D**) Effects of liposomal and free clodronate on the viability of MG-5 cells. The number of viable MG-5 cells significantly decreased after exposure to both forms of clodronate. (**E**) Repeated immunostaining using CD11b and PI antibodies showing the time course of death of MG-5 cells after exposure to both forms of clodronate. Scale bar, 200 μm. Data are presented as the mean ± SEM.

Although free clodronate does not easily enter into microglia because of difficulty in passage through cell membranes, it slowly accumulates in cells that remain in the surrounding medium [37]. In addition, recent studies have suggested that free clodronate could induce apoptosis of microglia [38-40]. Therefore, we examined two forms of clodronate, free and liposomal, for their ability to eliminate microglia using microglial cell line MG-5 [32]. These cells were exposed to different concentrations of free or liposomal clodronate, and PI staining was performed to evaluate MG-5 cell death. Many PI-positive cells (indicating dead/dying cells) were observed 24 h after clodronate exposure (Figure 2A), and both forms of clodronate significantly increased the number of PI-positive cells in a concentration-dependent manner (Figure 2B). Three days after exposure, both forms of clodronate significantly reduced the number of MG-5 cells (Figure 2C, D). Although partial reduction was already observed using 5 μg/mL of liposomal clodronate, a nearly 10-fold higher concentration of free clodronate was required to produce a similar effect (Figure 2D). Most MG-5 cells were eliminated by 200 μg/mL of liposomal clodronate and 2,000 μg/mL of free clodronate (Figure 2D). Analyses of repeated immunostaining of the same sample confirmed that the efficacy of low-concentration liposomal clodronate was similar to that of high-concentration free clodronate (Figure 2E). These results indicate that both free and liposomal clodronate can potentially eliminate microglia and that liposomal clodronate more potently eliminates microglia than free clodronate.

### Liposomal clodronate selectively eliminates microglia from primary astrocyte cultures

**Figure 3:**
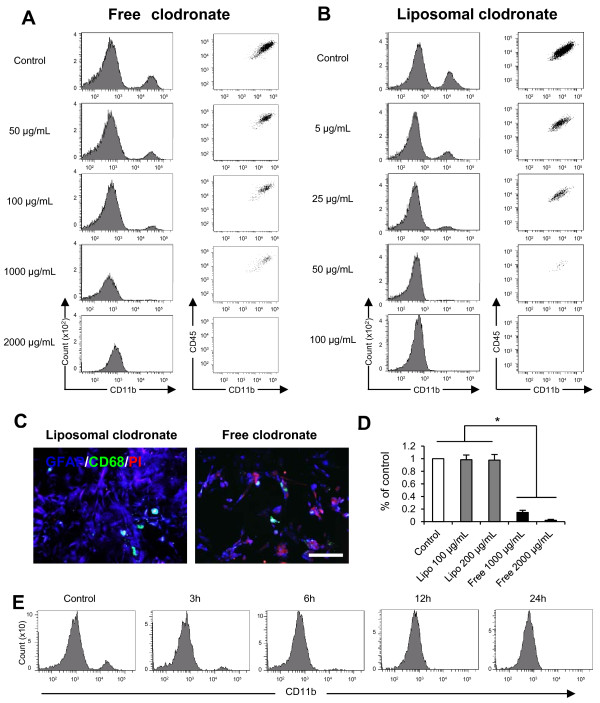
**Liposomal clodronate effectively eliminates microglia from primary astrocyte cultures.** (**A, B**) Flow cytometric analysis of primary astrocyte cultures 3 days after exposure to free (A) or liposomal clodronate (B). The result indicated dramatic reduction in the CD11 ^positive^ (left) and CD45^int^/CD11b^positive^/Gr-1^nega-int^ (right) populations in a concentration-dependent manner. Complete microglial elimination was achieved using 2,000 μg/mL of free clodronate and 100 μg/mL of liposomal clodronate. (**C**) Triple immunostaining of primary astrocyte cultures 24 h after exposure to each clodronate using GFAP (blue), CD68 (green), and PI (red) antibodies. There were many PI and GFAP double-positive cells after exposure to free clodronate. Scale bar, 200 μm. (**D**) Quantification of the number of GFAP-positive cells three days after exposure to free or liposomal clodronate (n = 5). The number of astrocytes significantly decreased after exposure to free clodronate (*P <0.05, Kruskal-Wallis test with Dunn’s multiple-comparison test). (**E**) The duration of exposure to liposomal clodronate required for complete microglial elimination from primary astrocyte cultures. Flow cytometric analysis demonstrated that microglial elimination was achieved 12 h after exposure to 100 μg/mL of liposomal clodronate. Data are presented as the mean ± SEM.

Similar to the results obtained in MG-5 cells, both forms of clodronate completely eliminated microglia from primary astrocyte cultures after three days of treatment (2,000 μg/mL of free clodronate and 200 μg/mL of liposomal clodronate; data not shown). Because MG-5 cells appear to be relatively resistant to apoptosis [32], primary microglia could be eliminated at concentrations lower than those required for MG-5 cells. Therefore, we attempted to determine the optimal concentration of both forms of clodronate for selective microglial elimination from primary astrocyte cultures. Although flow cytometric analysis showed that concentrations of free clodronate required to eliminate primary microglia were similar to those required to eliminate MG-5 cells (2,000 μg/mL, three-day exposure), liposomal clodronate eliminated microglia at concentrations lower than those required to eliminate MG-5 cells (100 μg/mL, three-day exposure) (Figure 3A, B). To assess whether free and liposomal clodronate selectively eliminate microglia, we examined the viability of microglia and astrocytes in primary astrocyte cultures after treatment with each clodronate. PI-positive microglia were observed 24 h after exposure to both forms of clodronate (Figure 3C), whereas PI-positive astrocytes were rarely observed after liposomal clodronate exposure. However, many PI-positive astrocytes were observed after exposure to free clodronate (Figure 3C), and this type of clodronate significantly decreased the number of astrocytes three days after exposure (Figure 3D). These results indicate that free clodronate but not liposomal clodronate significantly affects the viability of astrocytes; therefore, only liposomal clodronate was further examined for microglial elimination. Next, the duration of exposure to liposomal clodronate was assessed at the concentration achieving complete elimination of microglia (100 μg/mL) and 12 h of exposure to liposomal clodronate were sufficient for microglial elimination (Figure 3E). These results demonstrated that liposomal clodronate selectively eliminates microglia from primary astrocyte cultures without affecting the viability of astrocytes.

### Liposomal clodronate did not affect the activation or proliferation of astrocytes

**Figure 4:**
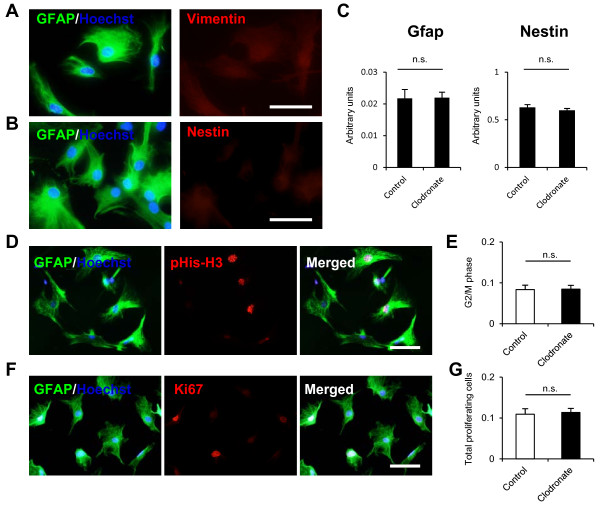
**Liposomal clodronate did not affect the proliferation or activation of astrocytes.** (**A, B**) Immunostaining of primary astrocyte cultures 12 h after exposure to liposomal clodronate using GFAP (green) and nestin (red; A) or vimentin (red; B) antibodies. Scale bar, 50 μm. (**C**) Gene expression levels of GFAP and nestin for astrocytes with/without liposome-clodronate exposure as determined by qPCR. Astrocytes isolated from each condition by flow-cytometry were used in these analyses. There were no significant difference in the gene expression of GFAP and nestin between the two (n = 5, Mann–Whitney *U* test). (**D-F**) Proliferation of astrocytes at six hours after the exposure was assessed by immunostaining using pHis-H3 (red; **D**) or Ki67 (red; **F**) antibodies Scar bar, 100 μm. Quantification of the proportion of p-His-H3-positive astrocytes (**E**) and Ki67-positive astrocytes (**G**) to total astrocytes after liposome-clodronate treatment (n = 5, paired Student’s *t*-test). Data are presented as mean ± SEM.

We next determined whether liposomal clodronate-mediated microglial elimination (100 μg/mL, 12 h) affects the activation or proliferation of astrocytes. After 12 h of exposure to liposomal clodronate, the morphological features of astrocytes did not significantly change and GFAP up-regulation was not observed (Figure 4A,B). Nestin and vimentin, representative markers of astrocyte activation, were not up-regulated in any astrocytes by exposure to liposomal clodronate (Figure 4A, B). To confirm these results, CD11b-negative population after 12 h of exposure was purified using flow cytometer, and qPCR was performed on FACS-purified CD11b-negative population. The gene expression of Gfap and nestin was not up-regulated in this population after exposure to liposomal clodronate compared to that without liposome clodronate exposure (Figure 4C). The proliferation of cells was assessed by the BrdU incorporation assay and Ki67 immunostaining. As shown in Figure 4D–G, the number of Ki67- and BrdU-positive proliferating cells among GFAP-positive astrocytes after exposure to liposomal clodronate did not decrease compared to that without liposomal clodronate exposure, suggesting that 100 μg/mL of liposomal clodronate did not inhibit the proliferation of astrocytes. These results demonstrated that liposomal clodronate does not affect the activation or proliferation of astrocytes during microglial elimination from primary astrocyte cultures.

### Liposomal clodronate eliminates microglia more effectively than other methods

**Figure 5:**
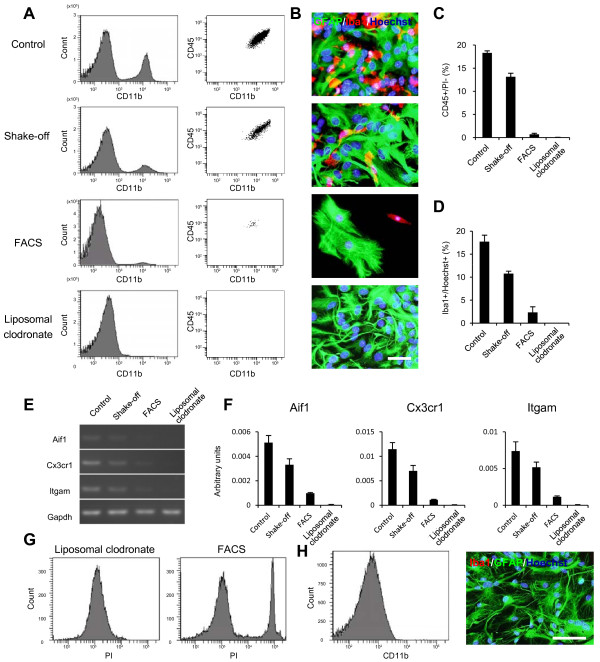
**Comparison of microglia-decontamination methods.** (**A**) Flow-cytometric assessment of enrichment of microglia in primary astrocyte cultures. Microglia (CD45^int^/CD11b^posi^/Gr-1^nega-int^) were not observed in primary astrocyte cultures after exposure to liposomal clodronate. (**B**) Immunostaining using Iba1 (red) and GFAP (green) antibodies. Scale bar, 50 μm. (**C**) The number of CD45-positive microglia in primary astrocyte cultures determined by FACS analysis (n = 6). (**D**) Quantification of the number of Iba1-positive microglia to the total number of cells in primary astrocyte cultures (n = 6). (**E, F**) Comparison of the gene expression of microglial markers such as Iba1, Cx3cr1, and Itgam (also known to CD11b) using semi-quantitative (**E**) and qPCR (**F**, n = 6). (**G**) The viability of astrocytes after exposure to liposomal clodronate or flow cytometric purification. There were few PI-positive dead cells 24 h after exposure to liposomal clodronate, but a large number of astrocytes were positive for PI 24 h after flow cytometric purification. (**H, I**) Flow cytometric analysis (**H**) and immunostaining (**I**) of astrocyte cultures 42 days after exposure to liposomal clodronate. Scale bar, 100 μm. Data are presented as the mean ± SEM.

We compared the efficacy of liposomal clodronate to that of previously reported methods in terms of microglial elimination [6,13,41]. Seven days after preparation of primary astrocyte cultures, microglial elimination was performed via exposure to liposomal clodronate, flow cytometric purification or the shake-off method, and the contaminating ratio of microglia was determined using flow cytometric analysis and immunostaining (Figure 5A–D). Flow cytometric analysis revealed that the conventional shake-off method significantly reduced the number of contaminating microglia, but a large number still remained (13.1 ± 0.8%, Figure 5A, C). Although effective microglial elimination was achieved by flow cytometric purification, a few microglia remained in the purified astrocyte cultures (0.7 ± 0.2%, Figure 5A, C). In contrast, no microglia were observed after exposure to liposomal clodronate (Figure 5A, C). Quantification of immunostaining by digital image analysis confirmed the efficacy of liposomal clodronate compared with the conventional shake-off method and flow cytometric purification (Figure 5B, D). These results were further confirmed using RT-PCR and qPCR. Although gene expression of Iba1, Cx3cr1, and Itgam (also known as Cd11b) was observed in primary cells after conventional shake-off and flow cytometric purification, their expression was not detected after exposure to liposomal clodronate (Figure 5E, F). In addition, many PI-positive cells were observed among the CD11b-negative population 24 h after flow cytometric purification (Figure 5G), suggesting that flow cytometric purification could affect the viability of astrocytes. Because primary astrocyte cultures are typically used for several months after their preparation [42,43], the microglia-eliminated cultures should be maintained over time. Immunostaining and flow cytometric analysis confirmed that the microglia-eliminated culture was stably maintained for eight weeks after exposure to liposomal clodronate (Figure 5H). These results demonstrated that liposomal clodronate is optimal for microglial elimination from primary astrocyte cultures compared to other methods.

### Delayed exposure to liposomal clodronate eliminates microglia from primary astrocyte cultures

Many authors have established various types of genetically modified astrocytes [29]. To examine the availability of delayed exposure to liposomal clodronate to these established culture lines, unpurified astrocyte cultures were exposed to liposomal clodronate eight weeks after preparation, in which more microglia exist than those at one week after the preparation (Figure 1L, M). Immunocytologic analysis performed three days after treatment revealed that 100 μg/mL of liposomal clodronate for 12 h eliminated microglia from unpurified cultures [see Additional file 2. These results indicate that this method can eliminate microglia from already established astrocyte cultures.

### Microglial contamination considerably affects the analytical results of primary astrocyte culture

**Figure 6:**
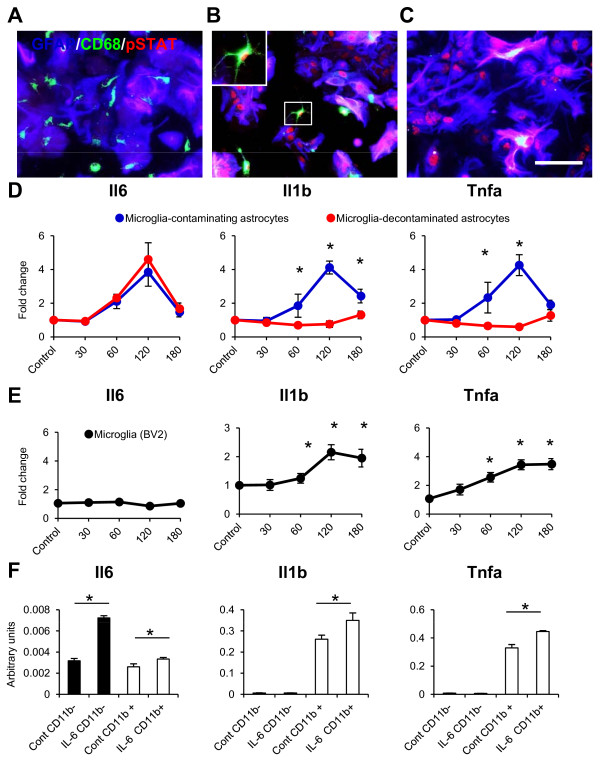
**Contaminating microglia rapidly produce TNF-α and IL-1β after IL-6 stimulation.** (**A–C**) Triple immunostaining using GFAP (blue), CD68 (green), and pSTAT3 (red) antibodies. Before IL-6 stimulation, pSTAT3 accumulation was rarely observed (**A**), but pSTAT3 accumulation was observed in microglia-contaminated (**B**) and microglia-eliminated astrocyte cultures (**C**) 1 h after IL-6 stimulation. pSTAT3 accumulation was observed in GFAP- and CD68-positive cells. Inset shows high magnification image of nuclear accumulation of STAT3 in microglia. Scale bar, 100 μm. (**D**) Time course of the gene expression of pro-inflammatory cytokines in microglia-contaminated or microglia-eliminated astrocytes after IL-6 stimulation (n = 5). (**E**) Time course of the gene expression of pro-inflammatory cytokines in BV2 cells after IL-6 stimulation (n = 3). (**F**) Gene expression of pro-inflammatory cytokines in FACS-purified CD11b-positive and CD11b-negative populations from conventional microglia-contaminated primary astrocyte cultures 1 h after IL-6 stimulation (n = 3). *P <0.05, Kruskal-Wallis test with Dunn’s multiple-comparison test (**D**, **E**), Mann–Whitney *U* test (**F**). Data are presented as the mean ± SEM.

Finally, we compared the temporal gene expression changes of pro-inflammatory cytokines after IL-6 stimulation in microglia-contaminated and microglia-eliminated primary astrocyte cultures. After 1 h of IL-6 stimulation, the nuclear translocation of pSTAT3 was observed in astrocytes and microglia (Figure 6A, B), suggesting activation of the JAK/STAT pathway in both astrocytes and microglia. In contrast, only GFAP-positive cells exhibited pSTAT3 accumulation in the nuclear region in microglia-eliminated astrocyte cultures (Figure 6C). qPCR demonstrated that IL-6, IL-1β and TNF-α expression was significantly up-regulated in microglia-contaminated cultures (Figure 6D). However, in microglia-eliminated cultures, only IL-6 expression was significantly up-regulated (Figure 6D). The time course of the gene expression of TNF-α and IL-1β in conventional primary astrocyte cultures was similar to that in the BV-2 microglial cell line (Figure 6E). These results suggest that rapid TNF-α and IL-1β gene induction in conventional primary astrocyte cultures after IL-6 stimulation was attributed to the activation of the JAK/STAT pathway in contaminating microglia. To directly confirm these results, conventional primary astrocyte cultures at 1 h after IL-6 stimulation were sorted into CD11b-positive and CD11b-negative populations by flow cytometer, and the gene expression of pro-inflammatory cytokines in each population was examined. qPCR analysis confirmed that IL-6 expression was upregulated in the CD11b-negative population and that TNF-α and IL-1β expression was upregulated in the CD11b-positive population (Figure 6F). These results highlight the importance of ensuring microglia elimination from astrocyte cultures to precisely clarify astrocyte biology.

## Discussion

Recent evidence has revealed the novel roles of astrocytes in CNS development and function [1-3,44,45]. Since astrocyte dysfunction has direct effects on neurodegenerative diseases, such as amyotrophic lateral sclerosis, Alzheimer’s disease, and Huntington’s disease [46], elucidation of astrocyte function under defined conditions can facilitate the development of new treatment strategies for CNS disorders [5,47,48]. Consequently, there is increasing interest in understanding astrocyte biology [1,5,48]. Although *in vivo* experiments have provided a considerable amount of information regarding the mechanisms of action [29,49-51], the complex cell–cell interactions and cytokine networks confound the interpretation of these results. In this regard, *in vitro* experiments are advantageous for investigating the biology of these cells because of the direct accessibility to components involved in a specific reaction. However, in primary astrocyte cultures, this advantage has been hindered by the difficulty in eliminating microglia and controlling astrocyte–microglia interactions [6]. Many authors have been concerned about the astrocyte culture system because microglial contamination, even though it was a small number of cells, could potentially affect astrocyte responses [52-54], confound the interpretation of results, and provide false information regarding astrocyte biology [6,11,12,55-58]. In fact, microglial contamination has been the cause of the incorrect assertion of production of nitric oxide and apolipoprotein E by astrocytes [6,30,55,59,60]. Although there are several methods to decrease the number of microglia in astrocyte cultures, no method has completely eliminated microglia from these cultures [6]. In this study, we demonstrated that exposure to liposomal clodronate completely eliminated microglia from primary astrocyte cultures, without affecting the viability, proliferation, or activation of astrocytes.

The most widely used technique for harvesting a single population of glia is dependent on a mechanical shake-off method based on the differential adherence properties of glia to the plastic of culture flasks/dishes [6,59]; astrocytes adhere more strongly to the plastic of culture flasks/dishes than other glia. Although repetitive shaking reduces the contaminating microglia on top of the monolayers, this method cannot achieve complete microglia elimination [6], especially when glia are strongly adherent to the plastic flasks/dishes or blanketed by astrocyte monolayers as shown in Figure 1B. As another method to reduce microglial contamination, anti-mitotic agents, such as 1-β-d-arabinofuranosylcytosine (Ara-C) have been employed to eliminate proliferating microglia in primary astrocyte cultures [61,62]. However, it is reported that low concentrations of Ara-C induces growth arrest and reactive responses in astrocytes while high concentrations kill astrocytes [63]. Because Ara-C is a potent S-phase–specific anti-tumor agent, it is likely to affect astrocytes, especially proliferating astrocytes [55]. Moreover, this agent only eliminates proliferating microglia and has little effect on non-proliferating microglia. l-Leucine methyl ester (LME), a lysosomotropic agent, has also been used to eliminate microglia, and effective reduction has been reported [56]. However, LME is toxic to astrocytes under some conditions because of its free diffusion into cells [43,55]. Indeed, it has been reported that LME unexpectedly affected the adhesion capacity [55] and reactivity of astrocytes [43,55].

Unlike previously used agents, liposomal clodronate selectively eliminated microglia without affecting the viability, proliferation or activation of astrocytes. This excellent result was obtained because of a strategy based on the different phagocytic activities of astrocytes and microglia. Although a high concentration of free clodronate affects the activities of cells via a non-specific extracellular mechanism, such as increasing their membrane permeability, clodronate is not a toxic molecule by itself in the extracellular environment [25]. Liposomes are also non-toxic and cannot freely move into cells [25]. Therefore, liposome-encapsulated clodronate rarely affects non-phagocytic cells [25]. In contrast, liposomal clodronate is readily absorbed by phagocytic cells, resulting in its rapid accumulation in the cytoplasm followed by the induction of apoptosis. Many authors have demonstrated that the liposome-mediated intracellular delivery of clodronate selectively eliminated macrophages without inducing toxicity in other cells [22,24,27,37]. Because microglia are resident CNS immune cells that are functionally equivalent to macrophages, their functions include phagocytosis and antigen presentation [64]. In the CNS, they not only clear cellular debris from the area in pathological conditions [53], but also continuously engulf tissue components in normal physiological conditions [16]. Indeed, their substantial phagocytic activity was confirmed *in vitro*, but phagocytic activity was not observed in astrocytes or other CNS cells [65].

Another strategy to reduce microglial contamination is cell surface marker-based sorting [13,41]. However, positive astrocyte selection cannot be performed because of the lack of a specific surface marker for astrocytes. In addition, although this method can effectively reduce the number of microglia from the primary astrocyte culture, this method requires specialized machinery, such as flow cytometric or magnetic cell sorting systems [13]. Therefore, this strategy is excellent but lacks versatility, resulting in its limited use. In contrast, our method requires only liposomal clodronate and, therefore, has broad utility for analyzing astrocyte biology. Furthermore, liposomal clodronate can eliminate human, rat and mouse macrophage-like cells [22,25]. The number of contaminating microglia in mouse primary astrocyte cultures is higher than that in other species [6], indicating that our method would be directly applicable to other species.

Neuroinflammation is a common pathological feature of the CNS, and astrocytes are strongly implicated in the initiation and development of neuroinflammation [13]. Therefore, many experimental studies are conducted to elucidate the molecular responses of astrocytes during neuroinflammation. In this study, we used microglia-eliminated cultures and found that astrocytes produced IL-6, but TNF-α and IL-1β, following the activation of JAK/STAT pathway. Since the up-regulation of the IL-6 gene in astrocytes may be because of the STAT3 function in a positive feedback loop to regulate gene expression, STAT3 may not be directly related to the pro-inflammatory properties of astrocytes. Conversely, all of these pro-inflammatory cytokines were up-regulated after IL-6 stimulation of microglia-contaminated astrocytes cultures due to the co-activation of contaminating microglia. These findings indicate the significance of our purified culture system to study neuroinflammatory responses of astrocytes. Our method will allow understanding of the functional responses of astrocytes to different inflammatory cytokines/chemokines without the complex effects of microglia and inflammatory networks present during neuroinflammation.

Although our method permits selective assessment for astrocytes, they are surrounded by microglia and their response is modified by microglia in the CNS [17]. Therefore, a detailed understanding of astrocyte-microglia cross-talk is also important to elucidate the disease onset and progression [44]. In this respect, astrocyte cultures with microglia offer an advantage over microglia-eliminated astrocyte cultures. In addition, microglial response also needs to be assessed without complex cell-cell interaction. In this study, we used immortalized microglia cell line BV-2 to assess microglial pro-inflammatory properties, their responses may not entirely reflect microglial responses in the CNS due to their partial failure in mounting immune responses while also revealing spontaneous activities [66]. Primary microglia will be useful to understand their inflammatory responses in the CNS. In the future, selective as well as interactive analysis for primary glial cells will provide new clues to treat CNS disease.

## Conclusions

In summary, our method for eliminating microglia from primary astrocyte cultures is simple, versatile and highly efficient. Because liposomal clodronate did not alter the activation or proliferation of astrocytes, these astrocyte cultures are immediately available for experiment, analysis, or further subpopulation sorting or culturing. Our method will help elucidate the reaction of astrocytes to a specific substance and the functional roles of astrocytes in physiological and pathological conditions, which will provide clues to understanding the role of astrocytes in neural disease, development and function. More careful analyses of the many functions of astrocytes in microglia-eliminated cultures is warranted to accurately understand astrocyte biology.

## Abbreviations

Ara-C, 1-β-d-arabinofuranosylcytosine; BrdU, 5-bromo-20-deoxyuridine; CNS, Central nervous system; DMEM, Dulbecco’s modified Eagle’s medium; FACS, Fluorescence-activated cell sorting; FBS, Fetal bovine serum; GAPDH, Glyceraldehydes-3-phosphate dehydrogenase expression; GFAP, Glial fibrillary acidic protein; Iba1, Ionized calcium-binding adaptor molecule 1; IL-6, Interleukin-6 IL-1β, interleukin-1β; JAK, Janus kinase; LME, l-leucine methyl-ester; PBS, Phosphate buffered saline; PE, Phycoerythrin; PFA, paraformaldehyde; PI, Propidium iodide; STAT, Signal transducer and activator of transcription; TNF-α, Tumor necrosis factor alpha.

## Competing interests

The authors declare that they have no competing interest.

## Authors’ contributions

HK designed studies, performed immuocytometric, flow cytometric, and gene expression analyses, and drafted the manuscript. HS supervised the overall project and performed flow cytometric analysis. KK performed qualitative assessments of microglia and gene expression analysis, and performed immuocytometric analysis. NvR supervised liposomal clodronate studies and assisted in manuscript preparation. KI supervised microglial studies and assisted in manuscript preparation. YI designed the studies and supervised the overall project. SO designed the studies, supervised the overall project, and performed the final manuscript preparation. All authors read and approved the final manuscript.

## Supplementary Material

Additional file 1 Primer list. Click here for file

Additional file 2 **Liposomal clodronate eliminates microglia from long-term cultured astrocytes.** At eight weeks after the preparation, primary astrocyte cultures were exposed with liposomal clodronate. (**A-B**) Double immunostaining using GFAP (green) and Iba1 (red) antibodies at three days after exposure to liposomal clodronate. (**A**) 100 μg/mL of liposomal clodronate completely eliminated microglia during three days of exposure. (**B**) 100 μg/mL concentration of liposomal clodronate completely eliminated microglia over 12 h of exposure duration. Scar bar, 500 μm. Click here for file
