# Supplementary material for: Liposomal clodronate selectively eliminates microglia from primary astrocyte cultures
Source: J Neuroinflammation. 2012 May 31;9:116. doi: 10.1186/1742-2094-9-116 (PMC3419615; doi:10.1186/1742-2094-9-116)
Supplement: Additional file 1 — Primer list. [file 1742-2094-9-116-S1.pdf]

Additional file 1

Gene symbol	Accession number	Size	5'- Forward primer -3'	5'- Reverse primer -3'
Gapdh	NM_008084.2	166	gacttcaacagcaactcccactct	ggtttcttactccttggaggccat
Nestin	NM 016701.3	183	gtcagctgagcctatagttcaacg	agagtcactcatcattgctgctcc
Gfap	NM 001131020.1	185	tgtactaacagagcgagcctatgc	gggacttgctgcctttaacattgg
Il6	NM_031168.1	169	gctctcctaacagataagctggag	ccacagtgaggaatgtccacaaac
Tnfa	NM_013693.2	153	ttatggctcagggtccaactctgt	tggacattcgaggctccagtgaat
Il1b	NM 008361.3	185	gggctggactgtttctaatgcctt	ccatcagaggcaaggaggaaaaca
Aif1	NM 019467	122	caaagaacacaagaggccaactgg	ttccatgctgctgtcatcagaagc
Cx3cr1	NM_009987.3	155	gacagacagactgagtcatatccc	agccagtgctattaaccactgagc
Itgam	NM 001082960.1	214	gtgaaaaatgagacgcctcctacc	ctttgccttggttttcactgtcgc

Additional file 1. Primer list
