# Supplementary material for: Liposomal clodronate selectively eliminates microglia from primary astrocyte cultures
Source: J Neuroinflammation. 2012 May 31;9:116. doi: 10.1186/1742-2094-9-116 (PMC3419615; doi:10.1186/1742-2094-9-116)
Supplement: Additional file 2 — Liposomal clodronate eliminates microglia from long-term cultured astrocytes. At eight weeks after the preparation, primary astrocyte cultures were exposed with liposomal clodronate. (A-B) Double immunostaining using GFAP (green) and Iba1 (red) antibodies at three days after exposure to liposomal clodronate. (A) 100 μg/mL of liposomal clodronate completely eliminated microglia during three days of exposure. (B) 100 μg/mL concentration of liposomal clodronate completely eliminated microglia over 12 h of exposure duration. Scar bar, 500 μm. [file 1742-2094-9-116-S2.pdf]

Additional file 2

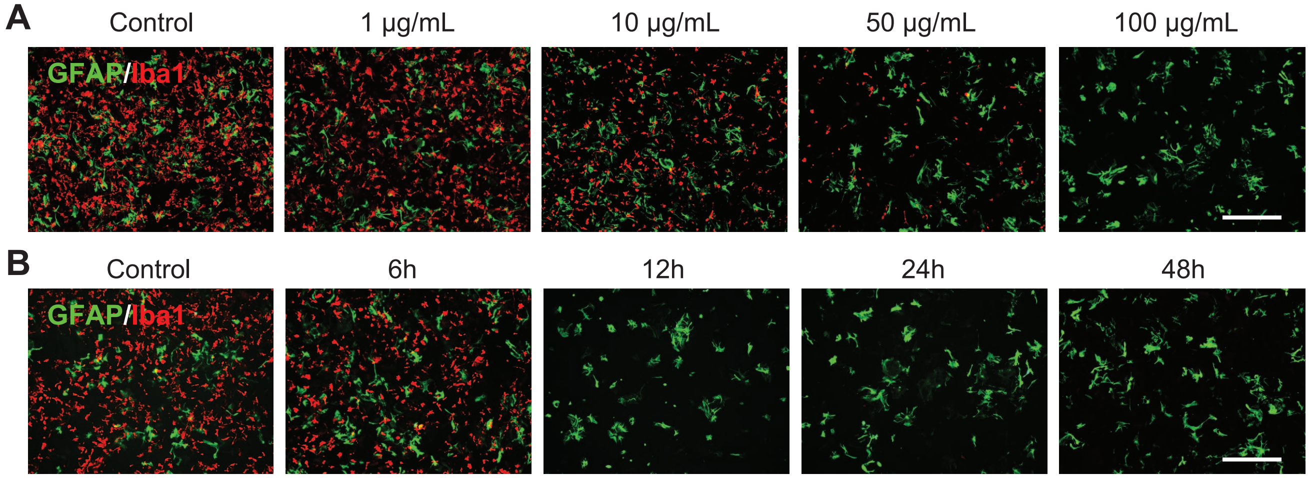

Additional file 2. Liposomal clodronate eliminates microglia from long-term cultured astrocytes.

At 8 weeks after the preparation, primary astrocyte cultures were exposed with liposomal clodronate. (**A-B**) Double immunostaining using GFAP (green) and Iba1 (red) antibodies at 3 days after exposure to liposomal clodronate. (**A**) 100 µg/mL of liposomal clodronate completely eliminated microglia during 3 days of exposure. (**B**) 100 µg/mL concentration of liposomal clodronate completely eliminated microglia over 12 h of exposure duration. Scar bar, 500 µm.
